# Interleukin-17A: The Key Cytokine in Neurodegenerative Diseases

**DOI:** 10.3389/fnagi.2020.566922

**Published:** 2020-09-29

**Authors:** Junjue Chen, Xiaohong Liu, Yisheng Zhong

**Affiliations:** Department of Ophthalmology, Ruijin Hospital, Shanghai Jiao Tong University School of Medicine, Shanghai, China

**Keywords:** interleukin-17A, neurodegenerative diseases, microglia, astrocytes, oligodendrocytes, glaucoma

## Abstract

Neurodegenerative diseases are characterized by the loss of neurons and/or myelin sheath, which deteriorate over time and cause dysfunction. Interleukin 17A is the signature cytokine of a subset of CD4^+^ helper T cells known as Th17 cells, and the IL-17 cytokine family contains six cytokines and five receptors. Recently, several studies have suggested a pivotal role for the interleukin-17A (IL-17A) cytokine family in human inflammatory or autoimmune diseases and neurodegenerative diseases, including psoriasis, rheumatoid arthritis (RA), Alzheimer’s disease (AD), Parkinson’s disease (PD), multiple sclerosis (MS), amyotrophic lateral sclerosis (ALS), and glaucoma. Studies in recent years have shown that the mechanism of action of IL-17A is more subtle than simply causing inflammation. Although the specific mechanism of IL-17A in neurodegenerative diseases is still controversial, it is generally accepted now that IL-17A causes diseases by activating glial cells. In this review article, we will focus on the function of IL-17A, in particular the proposed roles of IL-17A, in the pathogenesis of neurodegenerative diseases.

## Introduction

Interleukin-17A (IL-17A) is the signature cytokine of a subset of CD4^+^ helper T cells known as Th17 cells (McGeachy et al., 2019). The feature of Th17 cells is the expression of the RAR-related orphan receptor γ (RORγt) transcription factor (Ivanov et al., 2006). Initially termed CTLA8, IL-17A was cloned firstly in 1993 from a cDNA library of subtractive rodents and the IL-17-binding receptor was first reported in 1995 (Rouvier et al., 1993; Gaffen, 2011a). Researchers were interested because the sequences of the receptor and ligand of this molecule are different from those of other known cytokines in mammals (McGeachy et al., 2019). In human inflammatory or autoimmune diseases and neurodegenerative diseases, the IL-17A cytokine family has been reported to play a crucial function (Infante-Duarte et al., 2000; Luzza et al., 2000). IL-17A is produced by Th17 cells and other variable sources. In different conditions, the sources may be immune cells, including CD8+(Tc17) cells, “natural” Th17 cells, group 3 innate lymphoid cells (LC3s) and natural killer T (NKT) cells, or resident cells of the central nervous system (CNS), such as microglia (Cua and Tato, 2010).

Neurodegenerative diseases are characterized by progressive loss of selectively vulnerable populations of neurons, which progressively worsens over time and eventually leads to dysfunction (Hammond et al., 2019). There is a convincing body of evidence that protein aggregation, neuronal loss, and immune pathway dysregulation are common features of neurodegeneration (Hammond et al., 2019). These diseases include AD, PD, dementia with Lewy Bodies (DLB), multiple sclerosis (MS), and glaucoma. Glaucoma is characterized by visual field loss and progressive damage to the optic nerve axon and retinal ganglion cells (RGCs; Tian et al., 2015). The elevated intraocular pressure (IOP) is thought to be a major risk factor (Quigley and Broman, 2006; Wei and Cho, 2019). Studies have shown that IL-17A is involved in the pathogenesis of CNS neurodegenerative diseases. Levels of IL-17A in cerebrospinal fluid (CSF) and plasma are significantly increased in patients with MS, AD, and PD, and the expression levels are related to the severity and progress of diseases (Gu et al., 2013; Zhang et al., 2013; Kostic et al., 2014). Although the function of IL-17A in CNS neurodegenerative diseases is less understood and remains somewhat controversial, IL-17A is described to induce the occurrence and development of diseases by activating glial cells (especially microglia; Gu et al., 2013; Kolbinger et al., 2016). Therefore, for this review article, we mainly focused on recent studies on IL-17A and its role in neurodegenerative diseases.

## IL-17A and IL-17 Family Cytokines

There are six cytokines and five receptors in the IL-17 family (Gaffen, 2011b). The cytokines include IL-17A to IL-17F, and the receptors included IL-17RA to IL-17RE. These cytokines are dimer molecules, and they contain 163–202 amino acids with molecular weights ranging from 23 to 36 kDa (Gaffen, 2011b). The structures of these cytokines are similar to those of platelet-derived growth factor (PDGF) and nerve growth factor (NGF), which involve a special cystine knot fold architecture (Hymowitz et al., 2001). In the IL-17 family, IL-17A is the most studied cytokine, and it has the 57% sequence homology with the open reading frame 13 (ORF13) of Herpesvirus saimiri, a T cell tropic-herpesvirus that causes a lymphoproliferative syndrome (Gaffen, 2011b). Although they have a similar ORF13 sequence, the 3′ UTR of IL-17A has an adenylate-uridylate-rich (AU-rich) instability sequence, a common characteristic of growth factor and cytokine genes, and IL-17A can induce cytokine secretion in certain cells (Gaffen, 2011b). Thus, IL-17A is considered a cytokine (McGeachy et al., 2019). It has been shown that IL-17A exerts functions in the process of immune inflammation, neovascularization, and tumor development (Zhu et al., 2016; Kuwabara et al., 2017).

IL-17B through IL-17F was discovered when researchers screened for the homologous genes of IL-17A. IL-17B has been reported to play an important role in cancer and inflammation. The proliferation and migration of gastric carcinoma cells are facilitated by IL-17B through activating mesenchymal stem cells *in vitro* (Bie et al., 2017b). The resistance to the treatment of paclitaxel in breast cancer is promoted by IL-17B through activation of the extracellular regulated protein kinases 1/2 (ERK1/2) pathway (Laprevotte et al., 2017). IL-17B exerts a dual function in the development and progression of inflammation. In mucosal inflammation, IL-17B plays an anti-inflammation role (Reynolds et al., 2015). In rat models with indomethacin-induced intestinal inflammation, however, IL-17RB levels are increased, and intraperitoneal injection of IL-17B promotes the migration of neutrophils in normal mice, indicating that IL-17B has a pro-inflammatory function (Shi et al., 2000; Bie et al., 2017a). The source of IL-17C is different from IL-17A as IL-17C is produced by different cells, such as epithelial cells (Ramirez-Carrozzi et al., 2011). A recent study has shown that the peripheral nerve neurons are protected by IL-17C, which acts as a neurotrophic cytokine, during Herpes simplex virus reactivation (Peng et al., 2017). Also, through the expression of antimicrobial peptides, chemokines, and pro-inflammatory cytokines, epithelial inflammatory responses are stimulated by IL-17C. Although IL-17C plays a proinflammatory role in a skin inflammation model induced by imiquimod, it has a protective function in colitis elicited by dextran sodium sulfate (Ramirez-Carrozzi et al., 2011). IL-17D is preferentially expressed in some tissues, such as adipose tissue and skeletal muscle, as well as some organs, including lung, heart, and pancreas (Starnes et al., 2002). IL-17D has some effect during inflammation, tumors, and viral infection. Stimulation of endothelial cells with IL-17D induces a classic pro-inflammatory cytokine response, including granulocyte-macrophage colony-stimulating factor (GM-CSF), IL-6, and IL-8 and the increased expression of IL-8 is dependent on nuclear factor B (NF-κB)-dependent (Starnes et al., 2002). Compared to wild-type animals, IL-17D^(−/−)^ mice showed a higher incidence of cancer and exacerbated viral infections, indicating that the expression of IL-17D after viral infection and tumors is essential for the protection of the host (Saddawi-Konefka et al., 2016). Moreover, IL-17D plays a role in tumors and virus surveillance mediated by NK-cells (Saddawi-Konefka et al., 2016). There are some differences between IL-17E (now called IL-25) and other family members of IL-17. IL-25 is associated with type 2 immune response marked by increased serum Immunoglobin E (IgE), IgG, and IgA levels as well as pathological changes in the gastrointestinal tract and lungs. In the digestive tract, IL-25 limits chronic inflammation and regulates type 2 immune response (Owyang et al., 2006). IL-25 induces IL-4, IL-5, and IL-13 gene expression (Fort et al., 2001). An early study indicated that IL-25 exerts an opposite function in the pathogenesis of organ-specific autoimmunity compared to IL-17A (Kleinschek et al., 2007). IL-17F and IL-17A are similar in terms of function and source. These two cytokines are not only the result of gene replication, as they are located next to each other on the same chromosome, but are also co-produced by Th17 cells (Waisman et al., 2015). Similar to IL-17A, IL-17F contributes to inflammatory responses and barrier surface protection (Puel et al., 2011).

## Receptors and Signaling Pathways of IL-17

There are five receptors (IL-17RA to IL-17RE) in the IL-17 receptor family and these receptors are composed of two chains (Waisman et al., 2015). Among these receptors, IL-17A and IL-17F bind to the same receptor, which is a heterodimer composed of IL-17RA and IL-17RC (Ely et al., 2009; Hu Y. et al., 2010). The heterodimeric receptor composed of IL-17RA and IL-17RC is express in CNS resident cells, such as microglia and astrocytes, as well as CNS endothelial cells (Kebir et al., 2007; Das Sarma et al., 2009). However, the expression of the IL-17 receptor expresses on neurons remains controversial. Early studies have shown that rat dorsal root ganglion neurons and mouse neural stem cells express IL-17 receptors (Li et al., 2013; Segond von Banchet et al., 2013). Recently, human PD- induced pluripotent stem cell (iPSC)-derived midbrain neurons (MBNs) have been described to express IL-17 receptors (Kawanokuchi et al., 2008; Sommer et al., 2018).

**Figure 1:**
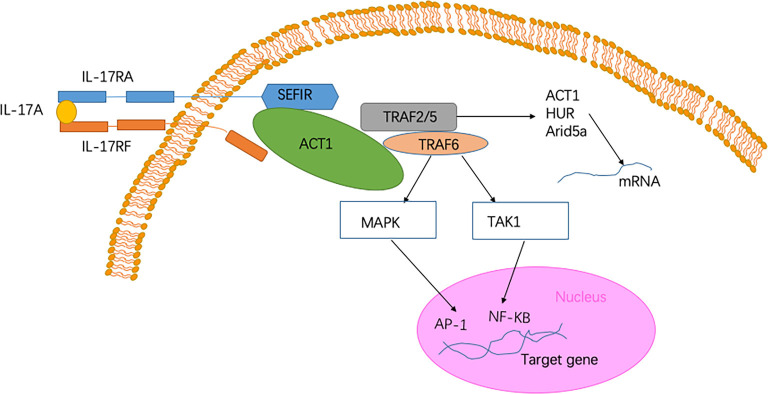
Signaling pathway of Interleukin-17A (IL-17A). The heterodimer receptor consists of two subunits, IL-17RA and IL-17RF, which bind to IL-17A, IL-17F, and IL-17AF ligands. The intracellular SEF/IL-17 receptor (SEFIR) domains interact with a corresponding SEFIR motif on the Act1 adaptor (Novatchkova et al., 2003). TNF-receptor associated factor 6 (TRAF6) and TRAF2/5 proteins bind to the TRAF-binding site in Act1. After binding to Act1, TRAF6 mediates the activation of the classical nuclear factor-κB (NF-KB) pathway of MAPK:AP-1. Collectively, these pathways trigger the transcriptional induction of target genes (Qian et al., 2007). In the IL-17 signaling pathway, a pathway of post-transcriptional mRNA stabilization is promoted through the recruitment of TRAF2 and TRAF5 by Act1 (Schwandner et al., 2000). This physiological process is achieved by controlling multiple RNA-binding proteins, such as HuR and Arid5a.

The IL-17 receptor family has one thing in common, namely, it shares a cytoplasmic motif termed “SEFIR” (SEF/IL-17 receptor; Novatchkova et al., 2003). After contact with IL-17 family cytokines and the IL-17R complex, Act1) an adaptor protein) is recruited to the SEFIR domain of the receptor complex (Qian et al., 2007; Liu et al., 2011; Waisman et al., 2015). The intracellular SEFIR domain then interacts with a corresponding SEFIR motif on the Act1 adaptor. Act1 then rapidly ubiquitinates another E3 ubiquitin ligase, namely TNF-receptor associated factor 6 (TRAF6; Schwandner et al., 2000; Qian et al., 2007). Ultimately, IL-17 signaling triggers the activation of the canonical NF-κB cascade response (Qian et al., 2007). Collectively, transcriptional induction of target genes is triggered by these factors (McGeachy et al., 2019; Figure 1). When the NF-κB cascade response is activated, IL-17- NF-κB signaling induces several positive and negative feedback circuits to control related physiological function. NF-κB upregulates the expression of B cell lymphoma 3-encoded protein (Bcl3) and then, in turn, facilitates the expression of multiple IL-17-NF-κB-driven anti-microbial and proinflammatory genes (Ruddy et al., 2004; Karlsen et al., 2010). However, IL-17- NF-κB signaling induces several negative feedback circuits to restrain the activation of NF-κB, such as deubiquitination (Garg et al., 2013; Cruz et al., 2017). Among the above signaling pathways, Act1 is an essential activator. The absence of the Act1 gene has been shown to cause complete failure in the response of cells to IL-17 (Qian et al., 2007; Liu et al., 2011).

## Function of IL-17A

Induction of the expression of chemokines, such as chemokine (C-X-C motif) ligand 1 (CXCL1), CXCL2, and CXCL8, is an important function of IL-17A. These chemokines can attract myeloid cells to injured or infected tissues (Onishi and Gaffen, 2010). IL-17A also induces the expression of IL-6 and granulocyte colony-stimulating factor (G-CSF), which promotes myeloid-driven innate inflammation (Gaffen et al., 2014). When encountering acute microbial invasion, IL-17A induces responses to protect the host. Overwhelming data suggest that IL-17A has a specific function in the prevention of Candida albicans. Antifungal immunity is regulated by IL-17A through upregulating antimicrobial peptides (e.g., defensins) and proinflammatory cytokines (e.g., CXCL1 and CXCL5; Conti and Gaffen, 2015; Drummond and Lionakis, 2019). The increased expression of proinflammatory cytokines and antimicrobial peptides has a synergistic effect on limiting fungal overgrowth (Conti and Gaffen, 2015; Drummond and Lionakis, 2019).

In injured psoriatic skin tissue, dysregulated IL-17 signaling promotes pathogenic inflammation. A phase 2 clinical trial has shown that inhibitory treatment of IL-17A is effective, indicating the pathogenic role of IL-17A in mediating important inflammatory pathways in psoriasis (Chiricozzi and Krueger, 2013). In ankylosing spondylitis (AS), another autoimmune disease, IL-17A has been shown to contribute to pathogenic inflammation. Two double-blinded phase-3 trials have reported that the use of secukinumab (an anti-IL-17A monoclonal antibody) to treat AS is effective (Baeten et al., 2015). However, researchers have failed to identify evidence of meaningful clinical efficacy with brodalumab (a human anti-IL-17A monoclonal antibody) treatment in rheumatoid arthritis (RA) at least when compared to treatment with methotrexate (Pavelka et al., 2015). Taken together, these data indicate that further studies are required to clearly understand the role of IL-17A in the pathogenesis of autoimmune diseases.

In healthy skin, commensal microflora induces the production of IL-17A, which provides anti-fungal protection (McGeachy et al., 2019). When injury destroys the epithelial barrier of the skin, IL-17A promotes epithelial-cell proliferation and can clear the pathogenic agents (Naik et al., 2015). Production of IL-17A from the local epithelium is driven by the microbiota, resulting in the anti-microbial function. Colonization with the segmented filamentous bacterium (SFB), a single commensal microbe, is sufficient to induce the production of IL-17A in the lamina propria of the small intestine of mice. SFB and Th17 cells mediate the protection from pathogenic microorganisms (Ivanov et al., 2009). A previous study has suggested that IL-17A is beneficial in controlling dysbiosis and maintaining a homeostatic balance in the gut. The predisposition to neuroinflammation is enhanced by abolishing the intestinal IL-17RA pathway, thus confirming the crucial role of the IL-17R pathway in mediating the protection of epithelial surface and interaction of host and microbiome (Ivanov et al., 2009; Kumar et al., 2016).

IL-17A promotes the repair of tissue. A crucial part of wound repair is the proliferation of epithelial keratinocytes. In keratinocytes, the expression of regenerating islet-derived protein 3-alpha (REG3A), an intestinal anti-microbial protein, is increased during psoriasis. IL-17A induces keratinocytes to express REG3A, and this process promotes the proliferation of keratinocytes after injury in psoriasis (Lai et al., 2012).

IL-17A and transcription factors that regulate adipocyte differentiation have been reported to act in concert to contribute to the suppression of adipogenesis (Ahmed and Gaffen, 2013). Mice with deficiency of both IL-17A and IL-17RA gain increased fat with age, and IL-17A suppresses the maturation of cells with adipogenic potential, indicating that IL-17A inhibits adipogenesis (Ahmed and Gaffen, 2013). In a healthy state, IL-17A directly influences the metabolic function of adipocytes. IL-17A produced by γδT cells controls the homeostasis of regulatory T cells and adaptive thermogenesis in adipose tissue (Kohlgruber et al., 2018).

These abovementioned findings show that IL-17A is not just an inflammatory factor. IL-17A usually protects the body during the acute injury, but when a wound takes a long time to heal and turns to a chronic injury, the effect of IL-17A may turn into erosion or hyperproliferation of the wound, ultimately leading to the loss of function (McGeachy et al., 2019).

## Role of IL-17A in Neurodegenerative Diseases

There are several divergent and shared pathological and clinical features of age-related CNS neurodegenerative diseases, such as diverse protein aggregation and selective vulnerability of the brain that impact the clinical presentation and immune responses of diseases (Hammond et al., 2019). Neurodegenerative diseases lead to impairments of a person’s memory and cognitive ability, and some of these diseases affect patients’ ability to speak, move, and breathe. Neurodegenerative disease is a multifactorial disease that included aging, mitochondrial defects, dysfunctions in autophagic lysosomal pathways, neurovascular toxicity, synaptic toxicity, accumulation of misfolded proteins, and liquid-phase transitions in pathological protein aggregation (Focus on Neurodegenerative Disease, 2018).

**Figure 2:**
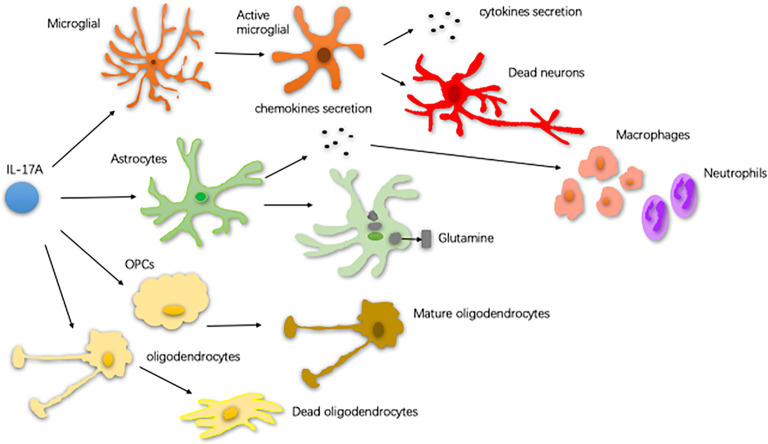
The way of glial cells respond to IL-17A. In central nervous system (CNS) neurodegenerative diseases, IL-17A binds to the receptor on the surface of microglia and activates microglia. Activated microglia secrete cytokines, exacerbating dopaminergic neurons loss. Astrocytes respond to IL-17A through generating chemokines to promote the recruitment of inflammatory cells, such as macrophages and neutrophils. IL-17A reduces the ability of astrocytes to absorb and transform glutamate as well as enhance the excitotoxicity of glutamate. IL-17A inhibits the maturation of oligodendrocyte lineage cells (OPCs) and exacerbates the TNF-α-induced oligodendrocyte apoptosis (Qian et al., 2007; Stromnes et al., 2008; Kang et al., 2010; Ji et al., 2013; Kang Z. et al., 2013; Liu et al., 2015; Rodgers et al., 2015; Liu Z. et al., 2019).

Neuroinflammation contributes, in part, to the occurrence of neurodegenerative diseases. Neuroinflammation in diseases, such as PD, AD, and ALS, is characterized by a reactive morphology of glial cells and increased levels of inflammatory mediators in the parenchyma (Ransohoff, 2016). To date, most pieces of evidence point to a pathogenic role for IL-17A in the CNS neurodegenerative diseases. IL-17A acts on multiple CNS resident cells to potentiate inflammation (Qian et al., 2007; Stromnes et al., 2008; Kang et al., 2010; Ji et al., 2013; Kang Z. et al., 2013; Liu et al., 2015; Rodgers et al., 2015; Liu Z. et al., 2019; Figure 2). It has been reported that IL-17A plays a regulatory factor in the induced cytokine network rather than as a direct role to mediate tissue damage during neuroinflammation (Zimmermann et al., 2013). Also, several studies have been reported the impact of medicinal plants on the level of IL-17A in neurodegenerative diseases (Table 1).

**Table 1:** The impact of medicinal plants on the level of interleukin-17A (IL-17A) in neurodegenerative diseases.

Authors	Disease	Medicinal plant	Effects on the level of IL-17A
Zhang Y. et al. (2015)	AD	Matrine	Reduction
Fragoulis et al. (2017)	AD	Kavalactones methysticin	Reduction
Chen et al. (2019)	AD	Oxymatrine	Reduction
Sanadgol et al. (2017)	MS	Ellagic acid	Reduction^a^
Zhang J. et al. (2015)/Wang et al. (2008)	MS	Tripchlorolide	Reduction^b^
Wang et al. (2012)	MS	Huperzine A	Reduction^c^
Zhao et al. (2011)	MS	Matrine	Reduction
Singh et al. (2007)	MS	Resveratrol	Reduction

*AD, Alzheimer’s disease; MS, Multiple sclerosis; ^a^Ellagic acid decreases IL-17A is dose-dependent; ^b^Tripchlorolide suppressed the mRNA levels of IL-17A; ^c^Huperzine A down-regulates mRNA and protein levels of IL-17A*.

AD is the most common type of late-onset dementia, and it is a complex molecular and genetic disease. The features of AD are neuronal and extensive synaptic loss, which leads to brain volume loss. Subsequently, the pathological changes of brain structure lead to a decline in patients’ memory and cognitive function that results in an inability to take care of themselves in daily life (Hammond et al., 2019). In recent years, the understanding of the pathological mechanism of AD has been constantly improved. The important pathological features of AD include intracellular neurofibrillary tangles resulting from the aggregation of hyperphosphorylated tau and deposition of extracellular neurotoxic plaques primarily composed of amyloid-β (Aβ; Holtzman et al., 2011). The aggregation of amyloid and tau eventually impacts the hippocampal, entorhinal cortex, and neocortical regions (Montine et al., 2012). Furthermore, Bussian et al. (2018) found that senescent microglia and astroglia influence neurofibrillary tangle formation and intraneuronal tau phosphorylation.

IL-17A may play a significant role in the pathogenesis of AD. In terms of clinical manifestations, elevated levels of IL-17A in plasma and CSF have been reported in patients with AD. For example, Chen et al. (2014) showed that IL-17A level in serum is increased in Chinese patients, and Hu W. T. et al. (2010) reported that the CSF level of IL-17A increases in patients. Also, Behairi et al. (2015) found that the baseline level of IL-17A is markedly higher in AD patients compared to controls. At the cellular and genetic level, there is also evidence of the correlation between IL-17A and the pathogenesis of AD. It has been reported that TH17 cell differentiation and activation as well as associated transcription factors are increased in patients with AD (Saresella et al., 2011). The induction and expression of IL-17A may be due to the polymorphism of Th17-related genes (Zota et al., 2009). BACE1 is a transmembrane asparty1 protease that plays a role in forming plaques in AD (Vassar, 2004). BACE1-deficient T cells have reduced IL-17A expression under Th17 conditions in AD mouse models (Hernandez-Mir et al., 2019). However, the effect of IL-17A on the pathogenesis of AD is controversial. Yang generated an AD mouse model with IL-17A overexpression and Yang et al. (2017) reported that IL-17A does not exacerbate neuroinflammation, and Yang also demonstrated that IL-17A overexpression decreases the level of soluble Aβ in the CSF and hippocampus as well as improves the metabolism of glucose (Yang et al., 2017). Two distinct human cohort studies have reported that the IL-17A level is decreased in AD patients compared to healthy controls (Doecke et al., 2012; Hu et al., 2013).

PD is the second most frequent form of neurodegenerative disease. PD is characterized by motor symptoms, including tremor, rigidity, and bradykinesia (Moustafa et al., 2016). A key pathological finding in PD is the aggregation of hallmark proteins (known as Lewy bodies), primarily composed of protein α-synuclein (Hamilton, 2000). There are certain routes for degeneration and aggregation of proteins generally spreading from the brain stem to the substantia nigra and other midbrain regions and then to the neocortex (Braak et al., 2004). Before the onset of symptoms, there is a massive loss of dopamine-producing neurons in the substantia nigra (Cheng et al., 2010). Recently, Sommer et al. (2018) have reported that T lymphocytes increase cell death in PD. iPSC-derived MBNs are mediated by IL-17A, indicating that IL-17A may be involved in PD pathogenesis. Liu et al. (2017) have demonstrated that Th17 cells infiltrated into the brain parenchyma through the disrupted blood-brain barrier (BBB) in PD. It has also been confirmed in animal experiments that IL-17A plays a role in the development of PD. Dopaminergic neurodegeneration, motor impairment, and BBB disruption are alleviated in mice with a deficiency of IL-17A (Liu Z. et al., 2019). However, the decreased plasma level of IL-17A is found in PD patients compared to controls (Rocha et al., 2018).

MS is an inflammatory demyelinating disorder of the CNS (Kostic et al., 2014), and susceptible genes and environmental factors are involved in disease pathogenesis. MS is characterized by the onset of recurring clinical symptoms followed by partial or total recovery. After 10–15 years of the disease, progressive deterioration is observed in up to 50% of untreated patients (Kolbinger et al., 2016). In approximately 15% of MS patients, the disease deteriorates from its onset (Gold et al., 2010). At present, the pathophysiology of MS has not been elucidated. MS may be primarily a neurodegenerative disease in which inflammation occurs as a secondary response that amplifies the state of progression (Kassmann et al., 2007). Compared to other neurodegenerative diseases, IL-17A has been mostly studied in MS. A possible pathogenic function of IL-17A in the pathogenesis of MS has been suggested. Kostic et al. (2014) demonstrated that the IL-17A level is increased in MS patients. In human MS brain tissue, the IL-17A-producing cells have been found but not in noninflamed brain tissue or normal white matter (Tzartos et al., 2008). In human MS plaques samples, an increase of IL-17A mRNA has been detected (Lock et al., 2002), and it has been reported that IL-17A content is related to BBB disruption and neutrophil expansion in CSF (Kostic et al., 2017). In terms of pathogenesis, Th17 cells may utilize the excitotoxicity of glutamate as an effector mechanism in MS. In MS, IL-17A is directly related to glutamate levels and may stimulate the Ca^2+^-dependent release of glutamate (Kostic et al., 2017). In the experimental autoimmune encephalomyelitis (an animal model of MS, EAE), IL-17RA expression is significantly increased in the CNS (Das Sarma et al., 2009). After binding to the IL-17RA complex in the CNS, IL-17A participates in the pathogenesis of EAE by promoting CD4 cell migration and secreting chemokines (Liu G. et al., 2014).

Amyotrophic lateral sclerosis (ALS) is another chronic neurodegenerative diseases of the CNS. The feature of ALS is the loss of upper and lower motor neurons, leading to motor and extra-motor symptoms. The neuropathological feature of this disease is the aggregation and accumulation of ubiquitylated proteinaceous inclusions in the motor neurons. In most subtypes of ALS, TAR DNA-binding protein 43 (TDP43) is the main component of these inclusions, but other abnormal protein aggregates are also present, including neurofilamentous hyaline conglomerate and misfolded superoxide dismutase (SOD1; Hardiman et al., 2017). The potential culprits of this disease may be the high-molecular-weight complexes that appear before protein aggregation, and the high-molecular-weight protein might contribute to the cell-to-cell spread of disease (Marino et al., 2015). Microglia have some impact on ALS disease. In the SOD1 mouse model of ALS, microglia have been found to contribute to the severity and progression of the disease. In contrast, microglial plays a neuroprotective role in the TDP43-dependent mouse model of ALS (Spiller et al., 2018). Together, these data indicate that microglial may exert different roles depending on the specific animal model and stimulus in ALS. In ALS, the IL-17A-mediated pathway may play a critical role. It has been reported that IL-17A serum concentrations in sporadic ALS and familial ALS patients are significantly higher than control subjects without autoimmune disorders (Fiala et al., 2010; Rentzos et al., 2010).

Glaucoma is also known as a neurodegenerative disorder characterized by RGCs death and axonal damage of the optic nerve, and ultimately leading to irreversible blindness (Levin et al., 2017). Glaucoma is considered as a disease caused by multiple factors, including high IOP mechanical injury, neurotrophic factor deprivation, ischemia/reperfusion injury, oxidative stress injury, excitatory glutamate toxicity, and abnormal immune-inflammatory response (Burgoyne, 2011; Rieck, 2013; Križaj et al., 2014). Studies have shown that immune dysfunctions, such as changes in cytokine signaling, immune cell proliferation, migration, and phagocytosis, as well as reactive gliosis, are common features of neurodegenerative diseases (Hammond et al., 2019). Autoimmunity is related to the pathogenesis of glaucoma as evidenced by large amounts of serum autoantibodies in glaucoma patients and animal models (Wax et al., 2008; Bell et al., 2013). In glaucoma, the elevated IOP is thought to be a major risk factor (Wei and Cho, 2019). However, increasing pieces of evidence have shown that the immune response plays a part in the pathogenesis of glaucoma. In recent years, some researchers have studied the IL-17A levels in patients with glaucoma. Yang et al. (2019) reported that the plasma levels of IL-17A are comparable in glaucoma patients and healthy people, and they demonstrated that the average frequencies of Th17 cells in patients with glaucoma is not significantly higher than that in the control group. In another study, however, researchers have demonstrated that the frequency of IL-17A-secreting cells and IL-17A^+^ CD4 T cells is significantly higher in patients with glaucoma than in controls (Ren et al., 2019). Using a retinal ischemia-reperfusion (IR) mouse model caused by acute elevated IOP, researchers have reported that elevated IOP increases the expression of IL-17A (Li et al., 2018). Because these studies measured IL-17A in peripheral blood in human patients and glaucoma is a complex disease whose pathogenesis has not been fully understood, further studies are needed to understand the role of IL-17A in glaucoma.

## The Role of IL-17A in a Different Model System of Neurodegenerative Diseases

Some animal models are used for the research of neurodegenerative diseases. In APP/PS1 mice, a transgenic mouse model of AD that overexpresses amyloid precursor protein (APP) with the Swedish mutation and exon-9-deleted presenilin 1, IL-17A is reported to play a key role in the induction and development of AD (Browne et al., 2013). IL-17A-producing T cell infiltrates in the brains of APP/PS1 mice which enhances the activation of glial cells and exacerbates neurodegeneration (McManus et al., 2014; Ahuja et al., 2017). IL-17A overexpression in this transgenic mouse model reduces soluble Aβ levels, decreases cerebral amyloid angiopathy, and improves glucose metabolism (Yang et al., 2017). IL-17A does not exacerbate neuroinflammation and significantly improves learning and anxiety deficits in this IL-17A overexpressing APP/PS1 mice (Yang et al., 2017). In 5XFAD mice, another animal model of AD, the production of IL-17A is decreased in the gut-residing immune cells (Saksida et al., 2018). In another animal models of AD including hAPP mice, 3xTg-AD mice, Mo/Hu APPswe PS1dE9 mice and Aβ1–42-Induced AD rat model, the level of IL-17A shows a significant upregulation compared with wild type mice (Jin et al., 2008; Zhang et al., 2013; Zhang Y. et al., 2015; Chen et al., 2015, 2019; Yang et al., 2015; St-Amour et al., 2019). In MPTP (1-methyl-4-phenyl-1,2,3,6-tetrahydropyridine) treaded mouse and MPP+ (1-methyl-4-phenylpyridinium) treated rats (animal models of PD), the levels of IL-17A are upregulated in the substantia nigra, spleen, serum, and mesenteric lymph nodes. Furthermore, IL-17A promotes neurodegeneration in PD depending on microglial activation and partly TNF-α release (Huang et al., 2014; Dutta et al., 2019; Liu Z. et al., 2019). The pathological role of IL-17A in EAE, an animal model of MS, has been demonstrated. A monoclonal anti-IL-17A Ab (MM17F3) autovaccination is reported to prevent histological and clinical manifestations of EAE (Uyttenhove et al., 2007). In the EAE mouse model with TnC−/−, the reduced ability of Th17 cells to produce IL-17 is observed in spleens (Momčilović et al., 2017). Also, IL-17A mRNA and protein levels increase in the cup-induced mouse model of MS (Sanadgol et al., 2017). For ALS, the SOD1G93A mice model is established and in this model, IL-17A is found to gradually increase with aging (Noh et al., 2014).

## The Relationship Between IL-17A and Glial Cells in Neurodegenerative Diseases

Microglial cells are an important part of the glial population of the CNS, and they account for approximately 10% of the total number of cells and are the largest number of mononuclear phagocytes in the CNS (Colonna and Butovsky, 2017). Microglial cells play a key role in CNS development and maintenance of CNS homeostasis. During CNS injury, microglial cells play a neuroprotective role by morphologically changing, proliferating, and migrating to the damaged site to phagocytose and eliminate microbes, protein aggregates, and dead cells (Colonna and Butovsky, 2017). Also, microglial cells secrete many soluble factors, such as neurotrophic factors and neurotrophic factors, which are involved in the immune response of the CNS. Aberrations in the normal phenotype or functions of microglial cells may lead to excessive synapse loss, contributing to the pathogenic mechanism of neurodegenerative diseases of the CNS. For example, microglial cells are induced to engulf neurons by recognizing phosphatidylserine exposed on tau-laden neurons, produce nitric oxide, and release of the MFGE8 opsonin. MFGE8 is required for engulfment and uptake of neurons (Brelstaff et al., 2018). Molecules expressed on the surface of microglial cells, such as LRRC33 and TREM2, affect relevant cellular pathways by binding to specific proteins (Qin et al., 2018; Li et al., 2019). These biological processes play a role in the pathogenesis of neurodegenerative diseases.

Although the specific mechanism of IL-17A in neurodegenerative diseases is still controversial, it is generally accepted that IL-17A causes diseases by activating glial cells (especially microglia). In a PD model, IL-17A activates microglia *in vitro* and accelerates the death of dopamine neurons through activating microglial cells (Liu Z. et al., 2019). Consistently, the IL-17A effect is abrogated after inhibition of the IL-17RA signaling pathway in microglia (Liu Z. et al., 2019), confirming the pathogenic relevance of microglial cells in mediating neurodegeneration in PD. Compared to controls, the expression of IL-17RA is increased in microglial cells of the CNS in EAE mice, which may be due to Toll-like receptors (TLR) signaling inducing IL-17RA expression in neuroglial cells (Liu G. et al., 2014). In EAE, IL-17A treatment induces the upregulation of chemokine secretion by microglial cells (Das Sarma et al., 2009). In aged rats, IL-17A participates in the process of neuroinflammation and cognitive impairment induced by lipopolysaccharide (LPS) through microglia activation (Sun et al., 2015). In acute glaucoma mouse models, inhibition of microglial activation reduces the secretion of IL-17A (Li et al., 2018). However, the relationship between IL-17A and microglia in neurodegenerative diseases has not been elucidated. Yang et al. (2017) reported that IL-17A overexpression in the mouse brain does not promote activation of microglia in AD mouse models. The evidence above suggests that further studies are needed.

Astrocytes play a central role in maintaining homeostasis of CNS, including regulation of synapse formation and maintenance, preserving neurological function, supplying energy to neurons, and maintaining the function of BBB (Pellerin and Magistretti, 2004; Molofsky and Deneen, 2015; Almad and Maragakis, 2018). Astrocytes are not homogeneous but can be specialized according to different regions of the CNS in which they reside (Pekny and Pekna, 2016). These glial cells of the CNS affect the structure and function of surrounding neurons. In the tripartite synapse, astrocytes can modulate synaptic activity by gliotransmission (Haydon, 2016). In the CNS, astrocytes communicate with surrounding neurons, microglia, and oligodendrocytes through hemichannels, which act in concert to maintain the normal function of CNS (Almad and Maragakis, 2018). During the disease course of the CNS, phenotypic conversion of microglial cells is induced by signals from astrocytes (Locatelli et al., 2018). An astrocyte that loses its function (termed A1 astrocyte) is induced by activated neuroinflammatory microglia (Liddelow et al., 2017). Taken together these data highlight the crucial role of astrocyte-microglia communication in neurodegenerative diseases of CNS.

**Figure 3:**
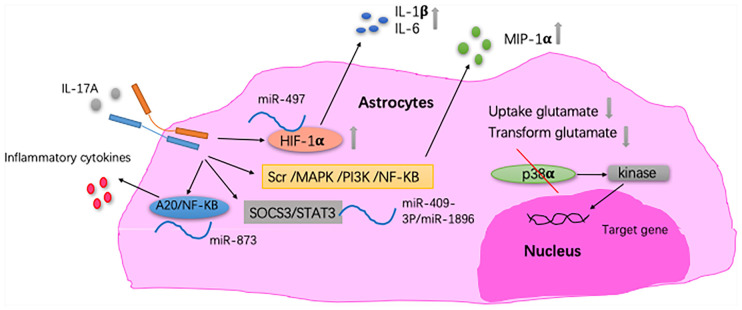
The relationship between IL-17A and astrocytes. In astrocytes, IL-17A induces expression of macrophage inflammatory protein-α (MIP-1α) through Src/MAPK/PI3K/NF-KB pathways (Yi et al., 2014). IL-17A enhances the excitotoxicity of glutamate by reducing the ability of astrocyte to absorb and transform glutamate (Kostic et al., 2017). In EAE mice, IL-17A triggers the downregulation of miR-497, thereby upregulating the hypoxia-inducible factor-1α (HIF-1α) transcription factor in astrocytes as well as IL-1β and IL-6 secretion by astrocytes. MiR-409-3p and MiR-1896 are involved in the process of IL-17A-mediated secretion of inflammatory cytokines by astrocytes by targeting the SOCS3/STAT3 signaling pathway in EAE mice. Under IL-17A stimulation, miR-873 participates in inflammatory cytokine production in astrocytes through the A20/NF-KB pathway in EAE mice (Liu X. et al., 2014; Shan et al., 2017; Liu X. et al., 2019). In EAE mice, proinflammatory gene expression induced by IL-17A is diminished through the abrogation of p38α in astrocytes, which was *via* the defective activation of MAPK-activated protein kinase 2 (Huang et al., 2015).

Astrocytes and IL-17A have been mainly studied in MS. One of the pathological features of MS is increased astrogliosis-associated neuroinflammation. Astrogliosis is a process by which astrocytes activate, proliferate, and upregulate the expression of the glial fibrillary acidic protein, and it is the main cause of MS plaque formation (Yi et al., 2014). By reducing the ability of astrocytes to absorb and transform glutamate, IL-17A enhances the excitotoxicity of glutamate (Kostic et al., 2017). Thus, astrocytes may act as a potential target for the neuroprotective effect of MS. In the CNS of EAE mice, the expression of IL-17RA is increased in astrocytes (Das Sarma et al., 2009; Colombo et al., 2014). Macrophage inflammatory protein-α (MIP-1α) is the β-chemokine that induces the directed migration of eosinophils, T lymphocytes and monoctyes, and it contributes to the pathogenesis of EAE. In primary astrocytes, IL-17A induces the expression of MIP-1α (Yi et al., 2014). Furthermore, miRNAs are involved in the pathogenesis mediated by IL-17A-expressing astrocytes in EAE (Liu X. et al., 2014, 2019; Shan et al., 2017). Under IL-17A stimulation, miRNAs participate in inflammatory cytokine production in astrocytes and, in turn, aggravate EAE development. Collectively, these findings suggest that the pathogenic role of the IL-17A-miRNA-astrocytes axis in EAE and may indicate a therapeutic target for treating MS. IL-17A blockade by Act1 ablation in astrocytes inhibits the induction of EAE and has a therapeutic effect (Kang et al., 2010; Yan et al., 2012). Also, in a mouse model of MS, proinflammatory gene expression induced by IL-17A is diminished through the abrogation of p38α in astrocytes (Huang et al., 2015; Figure 3). *In vitro* studies have shown that IL-17A secreted by activated astrocytes plays a neuroprotective role in acute neuroinflammation (Hu et al., 2014).

Oligodendrocytes are a group of glial cells in the CNS and have a supporting role for neuron migration, terminal differentiation, axon wrapping, axon recognition, myelin production, and myelin maintenance (Cai and Xiao, 2016). The main function of oligodendrocytes is the formation of myelin, which benefits nerve repair by maintaining myelin restoration (Bradl and Lassmann, 2010). Oligodendrocytes perform their physiological functions by communicating with neighboring astrocytes and neurons (Orthmann-Murphy et al., 2007). The loss or dysfunction of oligodendrocytes contributes to the vulnerability of the human brain to neurodegenerative diseases. For example, in AD and Huntington’s disease (HD), disturbances of myelin integrity are exacerbated compared to normal controls (Bartzokis et al., 2004, 2007). Increased numbers of oligodendrocyte progenitor cells (OPCs) are observed in ALS patients, indicating the failure of myelin regeneration (Kang S. H. et al., 2013). Recently, it has been reported that oligodendrocyte heterogeneity in human MS brain tissue may contribute to the disease progression (Jäkel et al., 2019). IL-17A plays a role in the development of oligodendrocyte lineage cells. IL-17A inhibits the maturation of oligodendrocyte lineage cells *in vitro* (Kang Z. et al., 2013) and it exacerbates the TNF-α-induced oligodendrocyte apoptosis (Paintlia et al., 2011). In EAE, mature oligodendrocytes and OPCs have different effects on the progression of the disease. Kang Z. et al. (2013) reported that the deletion of Act1, the adaptor protein required for IL-17 signaling, from mature oligodendrocytes does not affect the course of EAE. However, elimination of IL-17A signaling in OPCs (referred to as NG2 glia) reduces EAE severity.

## Conclusion

IL-17A is a signature of a key T helper cell population and evidence suggests a crucial role for IL-17A in the pathogenesis of autoimmune diseases and neurodegenerative diseases. The function of IL-17A has been proven to be varied as it not only contributes to pathogenic inflammation but also induces innate-like acute immune defenses. Thus, IL-17A is not simply an inflammatory factor. Although the specific mechanism of IL-17A in neurodegenerative diseases is still controversial, it is generally accepted that IL-17A causes diseases by activating glial cells. The functions of IL-17A have proven to be more adaptable and diverse than initially discovered. IL-17A may also play a key role in tissue damage. Our understanding of these processes is still lacking, particularly in the role of IL-17A in the pathogenesis of glaucoma. Also, we are still in the early stages of understandin how IL-17A interacts with different cytokines and how IL-17A signals are transmitted in response to microbial stimuli. Understanding how IL-17A interacts with different cells and cytokines is important. Uncovering the molecular pathways may allow the identification of better targets to modulate these cellular processes. Novel therapeutic strategies may be discovered by such studies.

## Author Contributions

JC wrote and edited the manuscript. XL and YZ edited the manuscript. All authors read and approved the final manuscript. All authors contributed to the article and approved the submitted version.

## Conflict of Interest

The authors declare that the research was conducted in the absence of any commercial or financial relationships that could be construed as a potential conflict of interest.

## Abbreviations

IL-17A: Interleukin-17A
Th cells: helper T cells
AD: Alzheimer’s disease
PD: Parkinson’s disease
MS: Multiple sclerosis
RORκt: transcription factor RAR-related orphan receptor gamma
NKT cells: natural killer T
LC3s: group 3 innate lymphoid cells
CNS: central nervous system
DLB: Dementia with Lewy Bodies
IOP: intraocular pressure
RGCs: retinal ganglion cells
CSF: cerebrospinal fluid
NGF: nerve growth factor
PDGF: platelet-derived growth factor
ORF13: open reading frame 13
iPSC: induced pluripotent stem cell
MBNs: midbrain neurons
TRAF6: TNF-receptor associated factor 6
NF-κB: nuclear factor κB
Bcl3: B cell lymphoma 3-encoded protein
AS: ankylosing spondylitis
RA: rheumatoid arthritis
IgE: immunoglobin E
IL: interleukin
SFB: segmented filamentous bacterium
REG3A: regenerating islet-derived protein 3-alpha
KKS: Kallikrein-Kinin System
Del-1: developmental endothelial locus-1
ICH: intracerebral hemeorrhage
BBB: blood-brain barrier
EAE: experimental autoimmune encephalomyelitis
TLR: Toll-like receptors
ALS: Amyotrophic lateral sclerosis
IR: ischemia-reperfusion
TNF-α: tumor necrosis factor-α
LPS: lipopolysaccharide
SERIF: SEF/IL-17 receptor
IL-17RB: IL-17 receptor B
ERK: extracellular regulated protein kinases
GM-CSF: granulocyte-macrophage colony stimulating factor
CXCL1: chemokine (C-X-C motif) ligand 1
G-CSF: granulocyte colony stimulating factor.
